# Pathology Characteristics of Lymphomas in Rwanda: A Retrospective Study

**DOI:** 10.24248/eahrj.v5i2.669

**Published:** 2021-11-15

**Authors:** Fiacre Mugabe Byiringiro, Felix Manirakiza, Déogratias Ruhangaza, Thierry Zawadi Muvunyi, Belson Rugwizangoga

**Affiliations:** a Department of Clinical Biology, School of Medicine and Pharmacy, University of Rwanda, Kigali, Rwanda; b Department of Pathology, University Teaching Hospital of Kigali, Kigali, Rwanda; c Department of Pathology, Butaro Cancer Center of Excellence, Burera, Rwanda; d Department of Pathology, Rwanda Military Hospital, Kigali, Rwanda

## Abstract

**Background:**

Lymphomas have been a global challenge for many decades and despite measures for prevention and management, the incidence continues to increase. There are two main categories, which are Non-Hodgkin's Lymphomas and Hodgkin's Lymphomas and most common etiologies are environmental, genetic alteration, radiation and some viruses.

**Objective:**

To describe pathology characteristics of lymphomas in Rwanda based on Hematoxylin and Eosin stained glass slides and immuno histo chemistry, and classify them according to clinical aggressiveness.

**Patients and Methods:**

We conducted a retrospective observational and descriptive study from January 2013 to December 2019. Lymphoma cases were retrieved together with relevant clinical and pathological information, and reviewed by independent pathologists. Histological diagnosis was classified according to the 2008 World Health Organization system in order to assign clinical aggressiveness of the lymphoma.

**Results:**

Three hundred and six lymphoma cases were enrolled. Males contributed to 57% of all reviewed case, and slightly over 50% were young aged ≤35 years. Approximately 191 (62%) of cases were nodal lymphomas. Approximately one fifth (18%) of lymphoma cases were HIV positive. Most 213(70%) cases were Non-Hodgkin's Lymphomas of aggressive forms 164(77%). Among 164 cases of aggressive Non-Hodgkin's Lymphomas, diffuse large B cell lymphoma was the leading subtype 91(55.5%), followed by solid lymphoblastic lymphoma 32(19.5%) and Burkitt lymphoma 17(10.4%). Among all Hodgkin lymphoma cases, 90(97%) were classical Hodgkin lymphomaof nodular sclerosis subtype. Hodgkin lymphoma patients were younger compared to Non-Hodgkin's Lymphomas patients (mean age of 24.78±16.3 years versus 38.6±22. 5years, *p=.000*).

**Conclusion:**

Substantial proportion of Lymphomapatients in Rwanda were also HIV positive. Interestingly, Non-Hodgkin's Lymphomas in Rwanda are predominated by the most aggressive forms, and these mostly affect a younger population. Optimal characterisation of such cases, using advanced methods, is recommended.

## BACKGROUND

Cancer is a burden worldwide with 17.5 million cases and more than 8.7million deaths in 2015.^1^ In Africa, between 2012 and 2030, the number of new cancer cases per year is expected to increase by 70%.^2^ In sub-Saharan Africa, more than 456,000 deaths were caused by cancer in 2012 and a double is predicted in 2030.^3^ Lymphomas are categorised in two main groups, Hodgkin lymphoma (HL) and non-Hodgkin lymphoma (NHL). In 2015, the global incidence of NHL and HL was 666,000 and 231,000 cases, respectively, while deaths were 78,000 and 24,000 cases, respectively.^1^

In Rwanda, a survey done for the period of 1979-1987 showed that NHL represented 83.5% of all lymphomas.^4^ The most recent epidemiological study on cancer in Rwanda, covering the period of 2000 to 2004 showed that lymphoma constituted 25.3% of all cancers and was predominated(79.8%)by NHLs.^5^ Thus, available data on the distribution of lymphomas in Rwanda are from 15 years back. Since then, huge changes in the diagnosis, classification and treatment of lymphoma have taken place around the world, thanks to the introduction of immunological and genetic testing among others. More specifically, in Rwanda, immunohistochemistry has recently been introduced for the confirmation and / or sub typing of lymphoma cases.

This study aimed at assessing the current clinical and pathological features of lymphoma in patients diagnosed with lymphoma in Rwanda on the basis of morphology and the newly introduced immunohistochemistry. Trends observed in the data generated will likely inform clinicians, researchers and healthcare administrators of areas to focus on in their interventions.

## METHODS

### Type and Setting of the Study

We conducted a retrospective descriptive study on lymphomas cases diagnosed in Rwanda during the period of 2013-2019. The main stay of this study was Butaro Cancer Centre of Excellence (BCCOE) which receives the vast majority of lymphomas cases in Rwanda for diagnosis and/or immunohistochemistry, as it is the main centre for cancer chemotherapy in the country.

### Data Collection

Data were obtained from BCCOE histopathology archives. Permanent hematoxylin and eosin (H&E) and immunoperoxidase-stained slides were re-evaluated for diagnosis confirmation by 2 independent pathologists. In case of divergent conclusions, the two pathologists made a consensus diagnosis. Cases were categorized into HL and NHL. We used the 2008 WHO classification^6^ to categorize the cases according to their clinical aggressiveness. The current 2016 WHO classification^7^ in which genomic profiling is an important component, couldn't be used in this study because genomic profiling is not yet incorporated in clinical services in Rwanda. Lymphoma staging data was not included as this information was missing from most files consulted.

### Data Management and Statistical Analysis

Demographic, clinical, morphology and immunohisto-chemistry data were entered into data collection sheets, transcribed into an excel sheets. The data were imported into and analyzed using statistical package for social sciences (SPSS) version 25 software (USA-Chicago by Norman H. Nie et al). Independent t-test was used for mean comparison, Fisher's exact test and Chi-square test used to compare proportions. A p-value <0.05 was considered for a significant statistical association between groups.

### Ethical consideration

Each and every case was given a code during data collection and matching it with the histopathology case number. No patient's name appeared neither on data collection form nor during data presentation. The study was approved by the University of Rwanda College of Medicine and Health Sciences (UR-CMHS) Institutional Review Board (IRB), ethical approval number “No452/CMHS IRB/2019”.

### Limitations of the study

We faced a challenge in staging all NHLs cases due to lack of clinico-radiological information in patients' files. Also, a probability of a low rate of detection of lymphomas in Rwanda because of culture and belief of some of the population.

## RESULTS

This study included 306 cases of lymphomas. There was a predominance of male patients 174(56.8%), as shown in Table1. Younger patients were most affected in that 166(54.2%) of patients were aged ≤35 years. The Southern region was more affected 77(25.2%) than other regions of Rwanda. HIV was positive in 25(18.4%) of patients with known HIV status (136 patients). The lymphoma disease was mainly nodal191(62.4%) *versus* 3115(7.6%) for extra-nodal lymphoma. Most specimens consisted of excisions 184(60.1%), with fewer incisions 69(22.5%), bone marrow biopsies 13(4.2%) and other core needle biopsies 40(13.1%). Cases were predominated 213(69.6%) by NHLs, while HLs represented 93(30.4%) of cases. Most 164(77.0%) NHLs were of the aggressive form, while indolent NHL was seen in only 46(21.6%) of NHL cases.

**TABLE 1:** Clinical and Demographic Characteristics of Patients

Characteristics	N	%
**Sex**		
Male	174	56.9
Female	132	43.1
**Age (mean=34.4)**		
<15	79	25.8
16-35	87	28.4
36-55	75	24.5
>55	65	21.2
**Residence**		
Kigali	53	17.3
North	58	19.0
South	77	25.2
East	31	10.1
West	57	18.6
Foreigners	30	9.8
**HIV Status**		
Positive	25	18.4
Negative	111	81.6
**Site of biopsy**		
Extra nodal	115	37.6
Nodal	191	62.4
**Biopsy procedure type**		
Bone marrow	13	4.2
Core needle	40	13.1
Excisional	184	60.1
Incisional	69	22.5
**Type of lymphoma)**		
*Non-Hodgkin lymphoma*	213	69.6
*Hodgkin lymphoma*	93	30.4

The histological typing of lymphomas shows that diffuse large B-cell lymphoma (DLBCL) 91(55.5%), lymphoblastic lymphoma (solid) 32(19.5%) and Burkitt lymphoma 17(10.4%) predominated the aggressive form of NHL, while 23(50%) of indolent NHLs consisted ofsmall cell lymphocytic lymphoma/chronic lymphocytic leukemia (SLL/CLL), as shown in Table 2. On the other hand, nodular sclerosis 40(45.6%) and mixed cellularity 36(40.9%) classical HL comprised the majority of HL cases.

**TABLE 2:** Histological Types and Aggressive Forms of Lymphomas

Subtypesof Hodgkin lymphoma and non-Hodgkin lymphoma	n	%
**Classical Hodgkin lymphoma**	**90**	**96.8**
Lymphocyte depleted 1CHL	6	6.8
Lymphocyte rich CHL	6	6.8
Mixed cellularity CHL	36	40.9
Nodular Sclerosis CHL	40	45.5
**Non-classical Hodgkin lymphoma**	**3**	**3.2**
Nodular lymphocytes predominant	3	3.2
**Indolent non-Hodgkin lymphoma**	**46**	**21.6**
Cutaneous T-cell lymphoma (mycosis fungoides)	3	6.5
Follicular lymphoma grade 1 & 2	5	10.9
Marginal zone lymphoma/mucosa-associated lymphoid tissue lymphoma	15	32.6
Small cell lymphocytic lymphoma & chronic lymphocytic leukemia	23	50.0
**Aggressive non-Hodgkin lymphoma**	**164**	**77**
Anaplastic large cell lymphoma	6	3.7
Burkitt lymphoma	17	10.4
Diffuse large B-cell lymphoma	91	55.5
Lymphoblastic lymphoma (solid)	32	19.5
Mantle cell lymphoma	3	1.8
Natural killer T cell lymphoma	2	1.2
Peripheral T-celllymphoma	3	1.8
Plasmablastic lymphoma	10	6.2
**Unclassified non-Hodgkin lymphoma**	**3**	**1.4**

^1^Classical Hodgkin's lymphoma

Correlative analysis (Table 3) shows that the neither sex (Fisher's exact test *P*=.301) nor HIV status (Fisher's exact test *P*=.143) influenced the distribution of the major types of lymphomas (HL and NHL). Age distribution between HL and NHL differed in that the proportion of HL cases decreased with age while that of NHL increased with age (Chi-square test *P*=.000). Moreover, there is a male predominance (81%) among aggressive NHLs, most of whom are <15 years of age (data not shown). NHLs shows an approximately equal distribution among nodal and extra-nodal sites, while the vast majority of HL affect nodal sites (Fisher's exact test *P*=.000).

**TABLE 3:** Clinico-Pathologic Characteristics of Lymphomas

Parameter	Characteristics	HL (n)	NHL (n)	*P-value^a^*
Sex (n=306)	M	57	117	0.301
	F	36	96	
Age group				
(years), (n=306)	<15	32	47	0.000
	16-35	37	50	
	36-55	20	55	
	>55	4	61	
HIV status	Positive	4	21	0.143
(n=136)	Negative	34	77	
Anatomical site	Nodal	85	106	0.000
(n=306)	Extra-nodal	8	107	

^a^Fisher's exact test (if only 2 groups) or Chi-square test (if more than 2 groups). HIV, human immunodeficiency virus; HL, Hodgkin's lymphoma; NHL, non-Hodgkin's lymphoma

## DISCUSSION

This study shows that lymphomas affects predominantly male than female population in Rwanda; that trend is almost similar for both NHL and HL (*P*=.301). In comparison, these results are similar to those reported from Egypt^8^, Iran^9^ and India.^10^ A peak age of HL between 15 to 35 years noticed in this study was also previously reported in other settings.^11^ However, there is lack of the second HL peak age in Rwanda, most likely due to the fact that there is a small number of the population above 65 years of age in Rwanda.^12^ In our cohort, the rate of HIV positivity among lymphoma cases was 18%, which is quite higher than the prevalence (3%) of HIV positivity reported in the general population of Rwanda.^13^ This finding was observed elsewhere.^14^

The predominance 213(70%) of NHLs (*versus* HL) observed in this study was also documented in studies done in Iran (64.5%), Iraq (76%) and Southern India (78.7%).^15–17^

Aggressive form of NHLs was over-represented in Rwanda compared to other settings. In accordance, while aggressive NHL represents 164(77%) of NHL in Rwanda, it is approximately 53% in Central and Southern America, 37% in Northern America and 36% in Poland.^18^ Moreover, the aggressive form of NHL in Rwanda exhibits male predominance (81%) and the most affected age range was <15 years. Similar albeit weaker trend was seen in a study done in Iran witha male to female ratio of 1.8:1.^15^

A possible predominance of paediatric forms of lymphoma (Burkitt and Burkitt-like, potentially misclassified) among aggressive NHL in Rwanda should be investigated using advanced methods as put forth in the current WHO classification;^19^ such methods merit to be introduced in Rwanda.

## CONCLUSIONS

As reported elsewhere, there is a male predominance and high rate of HIV positive cases among lymphoma patients in Rwanda. Lymphoma in Rwanda affects younger patients than in other regions. Interestingly, the most aggressive forms of NHL predominate in childhood, which may imply that these cases are Burkitt or Burkitt-like lymphomas. This assumption is to be verified through the introduction of genomic and other advanced diagnostic methods in Rwanda.
